# Drosophila *domino* Exhibits Genetic Interactions with a Wide Spectrum of Chromatin Protein-Encoding Loci

**DOI:** 10.1371/journal.pone.0142635

**Published:** 2015-11-10

**Authors:** Kaitlyn Ellis, Chloe Friedman, Barry Yedvobnick

**Affiliations:** Biology Department, Emory University, Atlanta, Georgia, United States of America; CNRS, FRANCE

## Abstract

The Drosophila *domino* gene encodes protein of the SWI2/SNF2 family that has widespread roles in transcription, replication, recombination and DNA repair. Here, the potential relationship of Domino protein to other chromatin-associated proteins has been investigated through a genetic interaction analysis. We scored for genetic modification of a *domino* wing margin phenotype through coexpression of RNAi directed against a set of previously characterized and more newly characterized chromatin-encoding loci. A set of other SWI2/SNF2 loci were also assayed for interaction with *domino*. Our results show that the majority of tested loci exhibit synergistic enhancement or suppression of the *domino* wing phenotype. Therefore, depression in *domino* function sensitizes the wing margin to alterations in the activity of numerous chromatin components. In several cases the genetic interactions are associated with changes in the level of cell death measured across the dorsal-ventral margin of the wing imaginal disc. These results highlight the broad realms of action of many chromatin proteins and suggest significant overlap with Domino function in fundamental cell processes, including cell proliferation, cell death and cell signaling.

## Introduction

The Drosophila *domino* (*dom*) locus was initially characterized as a gene required for cell proliferation and viability, hematopoiesis and homeotic gene silencing [1,2]. The *dom* sequence predicts proteins of the SWI2/SNF2 class of DNA-dependent ATPases, implicating *dom* in gene regulation at the level of chromatin modification/nucleosome remodeling [2]. Domino proteins are widely expressed in embryos and imaginal tissues, and the sequence is highly conserved [2,3,4]. The locus has been associated with several functions, including exchange of phosphorylated histone H2Av as part of the Tip60 complex [3]. Tip60 is a lysine acetyltransferase, which acetylates histone and nonhistone proteins [5], and contributes to positive and negative gene regulation [6,7]. Dom function is also required in germline and somatic stem cells [8,9], and for repression of E2F responsive loci [10], Elongator complex target regulation [11] and Notch signaling [12,4,13,14]. Additionally, we previously described an RNAi-based, targeted screen for genetic modifiers of a *dom* wing phenotype [14]. This screen revealed several major classes of interaction, including genes regulating RNA function, transcription, cell growth, proliferation and autophagy. The wide array of functions associated with *dom* indicate that the locus may be involved with diverse chromatin factors, however the identification of these factors is incomplete. Here we have initiated a genetic scheme to identify additional chromatin associated proteins that have a role within the larger functional network that includes Dom. We analyzed a set of previously-described and also more newly-characterized loci that encode chromatin-associated proteins [15,16], for genetic interaction with *dom* at the wing margin. We likewise analyzed other members of the *SWI2*/*SNF2* gene family [17]. Our results indicate that loss-of-function (LOF) for many of these proteins elicits synergistic biological effects with *dom*, demonstrating that depression of *dom* function sensitizes the wing margin to changes in the activity of a substantial fraction of these chromatin components.

## Materials and Methods

### Drosophila strains

The *C96-domR* and *C96-Gal4* strains were described previously [18,14]. The remaining strains listed below were obtained from the Bloomington (BL) Stock Center. The BL strain numbers are indicated within parentheses: y[1] sc[*] v[1]; P{y[+t7.7] v[+t1.8] = TRiP.HMS00941}attP2 (33981), y[1] sc[*] v[1]; P{y[+t7.7] v[+t1.8] = TRiP.HMS00919}attP2 (33962), *y[1] v[1]; P{y[+t7.7] v[+t1.8] = TRiP.JF02132}attP2* (26234), *y[1] v[1]; P{y[+t7.7] v[+t1.8] = TRiP.JF02211}attP2* (31921), *y[1] v[1]; P{y[+t7.7] v[+t1.8] = TRiP.HMJ02079}attP40* (42514), *y[1] v[1]; P{y[+t7.7] v[+t1.8] = TRiP.JF02212}attP2* (31922), y[1] sc[*] v[1]; P{y[+t7.7] v[+t1.8] = TRiP.HMS00271}attP2 (33394), y[1] sc[*] v[1]; P{y[+t7.7] v[+t1.8] = TRiP.HMS00676}attP2 (32888), y[1] sc[*] v[1]; P{y[+t7.7] v[+t1.8] = TRiP.HMS00233}attP2 (33361), y[1] sc[*] v[1]; P{y[+t7.7] v[+t1.8] = TRiP.HMS00617}attP2 (33734), *y[1] v[1]; P{y[+t7.7] v[+t1.8] = TRiP.HMJ02055}attP40* (42491), *y[1] v[1]; P{y[+t7.7] v[+t1.8] = TRiP.JF02336}attP2* (26772), *y[1] v[1]; P{y[+t7.7] v[+t1.8] = TRiP.HMS02020}attP40* (40853), y[1] sc[*] v[1]; P{y[+t7.7] v[+t1.8] = TRiP.HMS00931}attP2 (33974), *y[1] v[1]; P{y[+t7.7] v[+t1.8] = TRiP.JF02015}attP2* (25993), y[1] sc[*] v[1]; P{y[+t7.7] v[+t1.8] = TRiP.HMS01054}attP2 (34580), y[1] sc[*] v[1]; P{y[+t7.7] v[+t1.8] = TRiP.HMS00051}attP2 (34069), y[1] sc[*] v[1]; P{y[+t7.7] v[+t1.8] = TRiP.HMS00634}attP2/TM3, Sb[1] (33043), y[1] sc[*] v[1]; P{y[+t7.7] v[+t1.8] = TRiP.HMC03937}attP40 (55250), *y[1] v[1]; P{y[+t7.7] v[+t1.8] = TRiP.HMC04001}attP40* (55314), y[1] sc[*] v[1]; P{y[+t7.7] v[+t1.8] = TRiP.HMS00607}attP2 (33725), y[1] sc[*] v[1]; P{y[+t7.7] v[+t1.8] = TRiP.HMS00076}attP2 (33666), *y[1] v[1]; P{y[+t7.7] v[+t1.8] = TRiP.HMS02334}attP40* (41937), *y[1] v[1]; P{y[+t7.7] v[+t1.8] = TRiP.HMJ21708}attP40* (53697), y[1] sc[*] v[1]; P{y[+t7.7] v[+t1.8] = TRiP.HMS01387}attP2 (34978), *y[1] v[1]; P{y[+t7.7] v[+t1.8] = TRiP.JF02129}attP2* (26231), *y[1] v[1]; P{y[+t7.7] v[+t1.8] = TRiP.JF02431}attP2* (27085), *y[1] v[1]; P{y[+t7.7] v[+t1.8] = TRiP.JF02231}attP2* (31940), *y[1] v[1]; P{y[+t7.7] v[+t1.8] = TRiP.JF02524}attP2* (29360), *y[1] v[1]; P{y[+t7.7] v[+t1.8] = TRiP.JF02732}attP2* (31960), y[1] sc[*] v[1]; P{y[+t7.7] v[+t1.8] = TRiP.HMS00628}attP2/TM3, Sb[1] (32845), y[1] sc[*] v[1]; P{y[+t7.7] v[+t1.8] = TRiP.HMS01142}attP2 (34665), y[1] sc[*] v[1]; P{y[+t7.7] v[+t1.8] = TRiP.HMS00586}attP2 (33708), y[1] sc[*] v[1]; P{y[+t7.7] v[+t1.8] = TRiP.HMS00683}attP2 (32894), y[1] sc[*] v[1]; P{y[+t7.7] v[+t1.8] = TRiP.HMS00050}attP2 (34520), y[1] sc[*] v[1]; P{y[+t7.7] v[+t1.8] = TRiP.HMS00684}attP2 (32895), y[1] sc[*] v[1]; P{y[+t7.7] v[+t1.8] = TRiP.HMS00587}attP2 (33709), y[1] sc[*] v[1]; P{y[+t7.7] v[+t1.8] = TRiP.HMS00829}attP2 (33891), y[1] sc[*] v[1]; P{y[+t7.7] v[+t1.8] = TRiP.HMS00301}attP2 (33419), y[1] sc[*] v[1]; P{y[+t7.7] v[+t1.8] = TRiP.HMS01254}attP2 (34908), y[1] sc[*] v[1]; P{y[+t7.7] v[+t1.8] = TRiP.HMS00585}attP2 (33707), y[1] sc[*] v[1]; P{y[+t7.7] v[+t1.8] = TRiP.HMS00302}attP2 (33420).

### Genetic interaction tests

Assays for genetic interaction between strains deficient in function for Dom and other chromatin proteins involved RNAi expression mediated via the *Gal4-UAS* system [19]. Crosses were performed at 22°C to adjust *Gal4* expression for detection of genetic interactions. We previously described a recombinant chromosome (*C96-domR*) that contains both a wing margin driver (*C96-Gal4*) and *UAS-dom* RNAi transgenes [14]. This chromosome produces a dominant, but partially-penetrant wing nicking phenotype at 22°C that was validated as a *dom* LOF phenotype. The phenotype is also sensitive to changes in expression of multiple components of the Tip60 complex that contains Dom protein [3], as well as various members of the Notch pathway [14]. Strains containing TRiP (*UAS*-regulated) RNAi transgenes directed against a set of chromatin-associated proteins were each crossed with the *C96-domR* strain. The offspring of these crosses contain *C96-Gal4*- directed expression of both *domR* and tester RNAi transgenes. Control crosses include *C96-domR* mated with *w* ^*1118*^ flies, for baseline penetrance of the *C96-domR* phenotype, and *C96-Gal4* mated with the tester TRiP strain. The latter cross is used to determine if any wing phenotypes derive from LOF for the TRiP strains alone. In most cases no *C96-Gal4* phenotypes were observed. Therefore, for many assays, genetic interaction could be measured by changes in the penetrance of wing nicking relative to *w* ^*1118*^ control crosses, either enhancement or suppression. The *C96-domR* x *w* ^*1118*^ crosses exhibited a range of penetrance from 26%-54%, where wings are scored as positive if they contain one or more anterior margin nicks [14]. Modifiers designated as enhancers or suppressors showed significant differences from control crosses run simultaneously, as determined by a chi-square test. In nearly all cases the significance was high (P< 0.001, see Tables 1 and 2). We express the percent of nicked wings in the experimental vs the control class as a ratio in Tables 1 and 2, where a value greater than 1 (control) is enhancement, and lower than 1 is suppression. Because the control % of nicked wings varied significantly across the experiments, the ratios do not reflect a ranked order of phenotypic severity. We scored all flies emerging from a minimum of eight vials.

**Table 1:** Identification of *dom* modifiers among 30 loci encoding chromatin proteins.

BL #	Name	Effect	Ratio	% E/C	N	*C96-Gal4*
3605	*w* ^*1118*^	-	1.0	**-**	>500	-
33981	*PCAF*	E	3.4	88.3/26	747	-
33962	*HP1c*	E	2.13	55.3/26	511	-
26234	*CC8*	E	1.68	91.6/54.4	596	-
31921	*JIGR1*	E	1.43	75.1/52.4	530	-
42514	*CC25*	E	1.42	72.7/51.7	478	-
31922	*CC20*	E	1.38	72.4/52.4	512	-
27085	*ERR*	E	1.32	57.2/43.3	696	-
33394	*RPS9*	E	1.26	65.2/52.4	893	-
32888	*CC24*	E	1.24	50.1/40.4	469	-
33361	*CC4*	E	1.24	49.9/40.4	503	-
33734	*CC7*	E	1.2	48.8/40.4	492	-
42491	*CC9*	E	1.18	55.9/47.4	462	-
26772	*CC15*	E	1.16	63.3/54.4	695	-
40853	*MAF-S*	S	0.53	24.9/47.4	473	-
33974	*RYBP*	S	0.52	21.1/40.4	318	-
25993	*CC28*	S	0.49	26.9/54.4	360	-
29360	*CC32*	S	0.48	25.1/52.5	506	-
34580	*NUP50*	S	0.3	7.7/26	672	-
34069	*CAF1*	E	3.85	100/26	473	+ (10%)
33043	*PCNA*	E	3.09	80.3/26	314	+ (1%)
55250	*ASF1*	E	2.72	70.6/26	260	+ (curling)
55314	*TOP1*	E	1.42	37/26	653	+ (curling)
33725	*RPD3*	S	0.27	7.1/26	339	+ (10%/curling)
33666	*HEL25E*	-	-	-	4	+ (100%)
41937	*CC31*	-	-	-	44	+ (100%)
53697	*SIR2*	-	-	-	487	+ (62%)
34978	*TRIP1* *	-	-	-	26	+ (lethal)
26231	*CC34*	NE	0.98	50.9/52.2	664	-
31940	*CC30*	NE	1.03	47.7/46	432	-
31960	*DSP1*	NE	1.01	26.3/26	623	-

Table 1 shows the Bloomington stock number, gene name and modifier effect on *C96-domR* wing phenotype as Enhancer (E), Suppressor (S), No Effect (NE), or could not be determined (-) for the 30 tested loci. The 23 strains listed as modifiers showed highly significant wing nicking differences from controls (P< 0.001, chi square test). Ratio represents the % nicked wings observed in experimental divided by the control *w* ^*1118*^ (% E/C) in crosses to *C96-domR* run simultaneously. N = # of wings scored. At least 500 *w* ^*1118*^ control cross wings were scored for each assay. *C96-Gal4* column shows results of control crosses to the 30 strains to determine LOF effects. Nine strains showed such effects (+).

*The *TRIP1* cross to *C96-Gal4* was 100% lethal, but a small number of offspring eclosed from the *C96-domR* cross.

**Table 2:** Identification of *dom* modifiers among 12 other *SWI2*/*SNF2* loci.

BL #	Name	Effect	Ratio	% E/C	N	*C96-Gal4*
3605	*w* ^*1118*^	-	1.0	**-**	>500	-
32845	*ISWI*	E	2.28	92.3/40	518	-
34665	*CHD1*	E	2.25	82.2/36.5	580	-
33708	*INO80*	E	1.86	67.9/36.5	558	-
32894	*XNP*	E	1.48	54.2/36.5	546	-
34520	*BRM*	E	1.21	44.3/36.5	467	-
32895	*HEL89B*	E	1.20	58.9/49.2	414	-
33709	*MARCAL1*	S	0.89	44/49.6	730	-
33891	*ETL1*	S	0.41	17.4/42.3	448	-
33419	*Mi-2* *	E	2.36	100/42.3	5	+ (13%/curling)
34908	*KISMET*	S	0.44	18.6/42.3	290	+ (7.5%)
33420	*CHD3*	NE	0.97	41/42.3	542	-
33707	*OKR*	NE	0.92	38.7/42.3	674	-

Table 2 shows the Bloomington stock number, gene name and modifier effect on *C96-domR* wing phenotype as Enhancer (E), Suppressor (S) or No Effect (NE) for the 12 *SWI2*/*SNF2* loci. The 10 strains listed as modifiers showed highly significant wing nicking differences from controls (8 strains P< 0.001, chi square test, *MARCAL1*: P< 0.003, Mi-2: P<0.007). Ratio represents the % nicked wings observed in experimental divided by the control *w* ^*1118*^ (% E/C) in crosses to *C96-domR* run simultaneously. N = # of wings scored. At least 500 *w* ^*1118*^ control cross wings were scored for each assay. *C96-Gal4* column shows results of control crosses to the 12 strains to determine LOF effects. Two strains showed such effects (+).

*Only 3 flies eclosed from the *Mi-2* x *C96-domR* crosses and 5 wings could be scored, all of which were scalloped severely (Fig 2). The *Mi-2* x *C96-Gal4* control crosses produced 45 offspring, where 12/90 wings contained minor nicks, and 100% were curled.

### Mounting of wings

Wings representative of the average phenotype for each of the strains were mounted onto a slide with Euparol and photographed using a light microscope [12]. The photographs were put in gray scale and sharpened using Adobe Photoshop.

### Wing disc antibody staining

Imaginal wing discs were dissected in 1X phosphate buffered saline (PBS), fixed for 20 min in 4% paraformaldehyde, and washed 3 times (1X PBS) prior to being permeablized with 0.3% Triton X-100 in PBS (PBST) for 20 min, and washed once more in 1X PBS [20]. The discs were then incubated overnight at 4°C with 10% normal goat serum (NGS) and primary antibody in 0.1% PBST. Subsequently, the discs were washed 5 times (0.1% PBST) and then incubated overnight at 4°C with NGS and secondary antibody in 0.1% PBST. After the discs were washed 5 more times, they were incubated overnight in n-propyl gallate in glycerol at 4°C, and prepared for confocal microscopy. Images were assembled with Photoshop software (Adobe). Cell death staining used Dcp-1 antibody (1:100, rabbit polyclonal; Cell Signaling) and Alexa 647 secondary antibody (1:100).

## Results

### Genetic interaction assays between *dom* and other loci encoding chromatin-associated proteins

The *C96-domR* strain constitutively expresses *UAS*-*dom* RNAi across the dorsal-ventral margin of the wing imaginal disc under *C96*-*Gal4* control, leading to a dominant and moderately penetrant nicked wing phenotype [14]. The *C96-Gal4* transgene contained in the *C96-domR* strain can simultaneously drive other *UAS*-regulated RNAi in transheterozygotes with *UAS-domR*, allowing tests for synergistic effects between the two loci. We investigated loci encoding proteins implicated by others in chromatin binding through various criteria, including the DamID technique [15,16]. This method requires that a tested polypeptide direct bacterial Dam (DNA adenine methyltransferase) close enough to DNA for adenine methylation to occur in a reproducible fashion at specific genomic regions. These investigators analyzed a set of previously defined chromatin proteins, as well as a new set of candidates. They chose candidate loci based on several criteria, including that the proteins contain a domain typically found in chromatin proteins, or alternatively that they interact with a known chromatin protein in a yeast two-hybrid screen. Their DamID study ultimately focused on 70 previously-described chromatin proteins, and 42 newly-characterized proteins that they named chromatin components (CC). We chose 30 strains to analyze, for which *UAS*-regulated TRiP RNAi strains were available from the Bloomington Drosophila Stock Center; 16 strains targeted new candidate CC loci and 14 targeted previously characterized loci encoding chromatin proteins. Three of the CC loci analyzed here are characterized loci not previously known as encoding chromatin components (*JIGR1*, *MAF-S*, *TRIP1*). Females of the *C96-domR* strain were mated to males of each of the 30 TRiP strains, and simultaneously to *w* ^*1118*^ control males. We also mated *C96-Gal4* driver strain females to males of the TRiP strains to determine if any wing margin effects were produced through LOF for the tester strain alone.

The results for these 30 crosses are summarized in Table 1. This table presents the data for the enhancers followed by the suppressors, first for the loci that did not exhibit a phenotype after the *C96-Gal4* control cross, followed by the loci that did show a phenotype after this cross. We found that 77% (23/30) of the TRiP strains produced either synergistic enhancement or suppression of the *C96-domR* wing nick phenotype. Six strains were suppressors (*MAF-S*, *RYBP*, *CC28*, *CC32*, *NUP50*, *RPD3*), while the remaining 17 were enhancers (*PCAF*, *HP1c*, *CC8*, *JIGR1*, *CC25*, *CC20*, *ERR*, *RPS9*, *CC24*, *CC4*, *CC7*, *CC9*, *CC15*, *CAF1*, *PCNA*, *ASF1*, *TOP1*). The fraction of modifiers detected in the set of new candidate chromatin loci (12/16 = 75%) vs the previously-characterized loci known to encode chromatin proteins (11/14 = 79%) was very similar. Nine of the strains exhibited a phenotype when crossed with *C96-Gal4* alone; phenotypes included wing nicking, wing curling, or near-complete lethality. For five of these nine the phenotypes were minor, allowing scoring of genetic interaction with *dom*, as enhancement (*CAF1*, *PCNA*, *ASF1*, *TOP1*), or suppression (*RPD3*). Four of the nine strains (*HEL25E*, *CC31*, *SIR2*, *TRIP1*) could not be scored for interaction with *dom* because of a highly-penetrant wing nick or lethal phenotype. Three strains (*CC34*, *CC30*, *DSP1*) did not display a phenotype via the *C96-Gal4* cross, and also showed no significant modification of the *C96-domR* phenotype.

**Fig 1:**
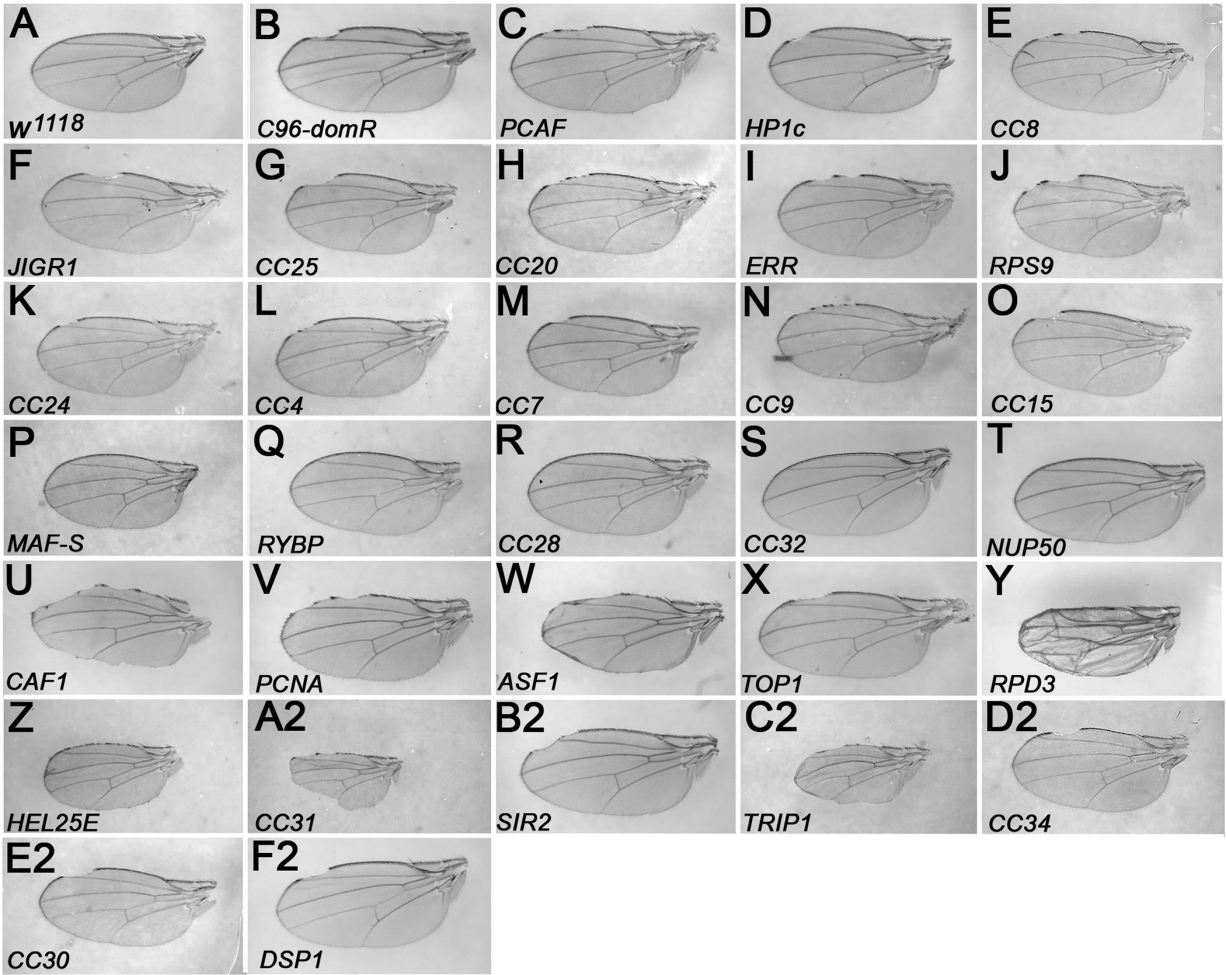
Genetic interactions of 30 chromatin protein encoding loci with *domino*. Wing mounts were prepared from the following Bloomington (BL) strains after crosses to *C96-domR*. Wings shown in panels C-F2 derived from crosses to TRiP strains. A. Wild Type wing BL3605 *w* ^*1118*^, B. Control wing *C96-domR/w* ^*1118*^, C. BL33981 *PCAF*, D. BL33962 *HP1c*, *E*. BL26234 *CC8*, *F*. BL31921 *JIGR1*, *G*. BL42514 *CC25*, *H*. BL31922 *CC20 I*. BL27085 *ERR*, *J*. BL33394 *RPS9*, *K*. BL32888 *CC24*, *L*. BL33361 *CC4*, *M*. BL33734 *CC7*, *N*. BL42491 *CC9*, *O*. BL26772 *CC15*, *P*. BL40853 *MAF-S*, *Q*. BL33974 *RYBP*, *R*. BL25993 *CC28*, *S*. BL29360 *CC32*, *T*. BL34580 *NUP50*, *U*. BL34069 *CAF1*, *V*. BL33043 *PCNA*, *W*. BL55250 *ASF1*, *X*. BL55314 *TOP1*, *Y*. BL33725 *RPD3*, *Z*. BL33666 *HEL25E*, *A2*. BL41937 *CC31*, *B2*. BL53697 *SIR2*, *C2*. BL34978 *TRIP1*, *D2*. BL26231 *CC34*, *E2*. BL31940 *CC30*, F2. BL31960 *DSP1*

Representative wings for the 30 crosses, and the *C96-domR*/*w* ^*1118*^ control are shown in Fig 1. In many cases, enhanced wings show much greater wing nicking penetrance, but otherwise resemble the *C96-domR*/*w* ^*1118*^ control class in terms of wing blade and margin structure (Fig 1, panels C-N). However, several of the strains that exhibited an effect with *C96-Gal4* alone showed a more severe, synergistic wing blade/margin loss phenotype (Fig 1, panels U, V, W) or a larger structural defect (panel Y) in combination with *C96-domR*. Three strains showed an effect on wing size (Fig 1, panels Z, A2, C2), and in these cases we could only recover a few adult offspring. These three strains could not be assayed for interaction since the *C96-Gal4* control cross phenotype was either 100% penetrant or lethal (Table 1).

### Genetic interaction assays between *dom* and other members of the SWI2/SNF2 family

Dom is a member of a family of chromatin modelers and shares the SWI2/SNF2 ATPase domain structure with a group of Drosophila proteins [17]. To investigate potential functional interactions of this larger family with *dom*, we also performed the wing assay with a set of 12 TRiP strains targeting SWI2/SNF2-encoding loci. The results for these 12 crosses are summarized in Table 2. Table 2 presents the data as for Table 1, for enhancers then suppressors, first listing the 10 loci that did not exhibit a phenotype after the *C96-Gal4* control cross, followed by the 2 loci that did show a phenotype after this cross. We observed that 83% (10/12) of the SWI2/SNF2 strains interacted with *C96-domR* as either enhancers (*ISWI*, *CHD1*, *INO80*, *XNP*, *BRM*, *HEL89B*, *Mi-2*) or suppressors (*MARCAL1*, *ETL1*, *KISMET*). Two of the interacting strains showed a phenotype when crossed with *C96-Gal4* alone (*Mi-2*, *KISMET*). The *OKR* and *CHD3* loci did not modify the *C96-domR* phenotype.

**Fig 2:**
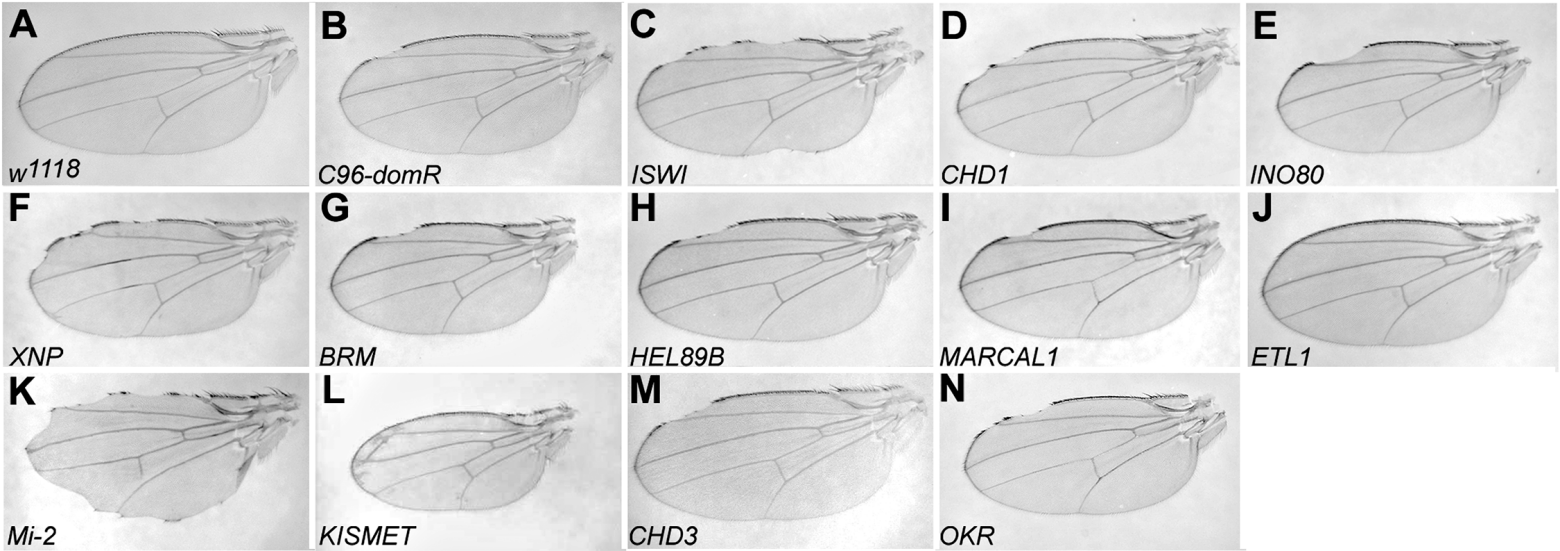
Genetic interactions of 12 SWI2/SNF2 protein encoding loci with *domino*. Wing mounts were prepared from the following Bloomington (BL) strains after crosses to *C96-domR*. Wings shown in panels C-N derived from crosses to TRiP strains. A. Wild Type wing BL3605 *w* ^*1118*^, B. Control wing *C96-domR/w* ^*1118*^, C. BL32845 *ISWI*, D. BL34665 *CHD1*, E. BL33708 *INO80*, F. BL32894 *XNP*, G. BL34520 *BRM*, H. BL32895 *HEL89B*, I. BL33709 *MARCAL1*, J. BL33891 *ETL1*, K.BL33419 *Mi-2*, L. BL34908 *KISMET*, M. BL33420 *CHD3*, N. BL33707 *OKR*.

Representative wings for the 12 SWI2/SNF2 crosses and controls are shown in Fig 2. We note that the *Mi-2* TRiP strain, which exhibited a weak wing nicking phenotype when driven alone via *C96-Gal4* (13%, Table 2), showed a strong synergistic enhancement of wing nick frequency and wing margin loss when crossed with *C96-domR* (Fig 2M). Moreover, very few flies survived this cross; 3 total flies eclosed from 8 vials, and only 5 wings could be unambiguously scored (Table 2).

### Cell death effects underlie a subset of *dom* genetic interactions

**Fig 3:**
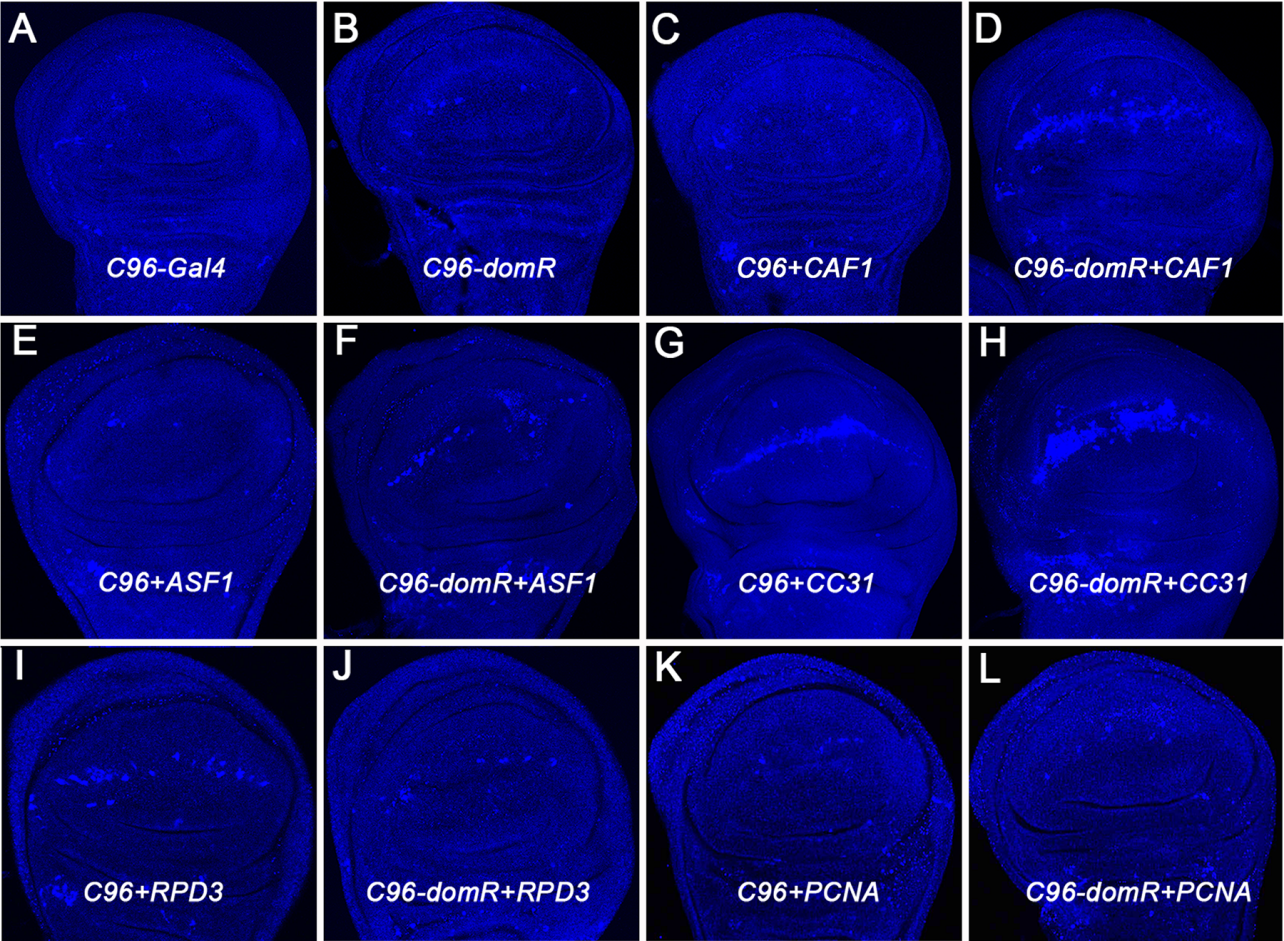
Cell death effects. Imaginal wing discs from following genotypes were stained with Dcp-1 antisera to measure caspase activity. A. Control *C96-Gal4/+* heterozygote shows occasional random stain, B. Control *C96-domR/+* heterozygote shows minor staining across dorsal-ventral wing margin, C. *C96-Gal4+Caf1*, D. *C96-domR+Caf1*, E. *C96-Gal4+ASF1*, F. *C96-domR+ASF1*, G. *C96-Gal4+CC31*, H. *C96-domR+CC31*, I. *C96-Gal4+RPD3*, J. *C96-domR+RPD3*, K. *C96-Gal4+PCNA*, L. *C96-domR+PCNA*.

The prior associations of *dom* LOF and cell death [1,9] suggested that some of the genetic interactions described above could result from synergism with an apoptosis pathway. Therefore, we examined a subset of modifier crosses by staining wing discs with cleaved Dcp-1 antibody (Fig 3), a marker for apoptosis [21]. Wing discs heterozygous for *C96-Gal4* exhibit occasional random spots of Caspase stain, whereas discs heterozygous for *C96-domR* show weak staining aligned with a portion of the dorsal-ventral wing margin (Fig 3A and 3B). When *C96-Gal4* is used to drive *CAF1* RNAi alone a weak Caspase stain is evident across the margin (Fig 3C), whereas the *C96-domR* + *CAF1* genotype leads to strong synergistic Caspase staining across the margin (Fig 3D). The extensive cell death evident across the margin reflects the striking loss of anterior and posterior wing margins observed in adult wings (Fig 1U). Similar analysis with the *ASF1* enhancer showed background levels of Caspase staining in the *C96-Gal4* + *ASF1* discs and moderately strong staining in *C96-domR* + *ASF1* discs (Fig 3E and 3F); again consistent with synergism evident in the adult wing phenotype (Fig 1W). *CC31* exhibited a small wing and 100% penetrant wing nicking phenotype when crossed with *C96-Gal4* alone (data not shown), or in combination with *C96-domR* (Fig 1A2); this precluded our ability to score for a genetic interaction in adult wings. However, we detected high levels of Caspase across the wing margin in *C96-Gal4* + *CC31* discs, and significantly higher levels of stain in *C96-domR* + *CC31* discs (Fig 3G and 3H), suggesting a synergistic effect. *RPD3* RNAi produced curled wings and exhibited nicks in 10% of the wings when driven by *C96-Gal4*; paradoxically, *RPD3* RNAi strongly suppressed the *C96-domR* wing nicking phenotype (Table 1). We compared Caspase stain levels from these crosses and observed strong staining in *C96-Gal4* + *RPD3* discs, but suppressed levels in *C96-domR* + *RPD3* discs (Fig 3I and 3J). We also note that adult wings derived from the latter cross exhibit an additional phenotype, as the dorsal and ventral wing surfaces are not adhered properly, leading to a disorganized structure (Fig 1Y). Therefore, the combined LOF for both *dom* and *RPD3* at the margin lead to more widespread effects. Finally, we compared Caspase stain levels in discs from *C96-Gal4* + *PCNA* and *C96-domR* + *PCNA* discs (Fig 3K and 3L). Despite showing strong enhancement of *C96-domR* wing nicking frequency (Table 1), *PCNA* RNAi does not lead to elevated Caspase stain levels in either genotype. Therefore, a subset of the genetic interactions we observe with *dom* can be measured in terms of cell death changes.

## Discussion

The initial molecular and genetic characterizations of *dom* linked it to chromatin remodeling and repression of homeotic loci, and showed a strong effect on cell viability and proliferation [1,2]. Subsequent studies demonstrated that *dom* plays a broad role during development and functions in both positive and negative regulation [12,4,13,10,14], at least in part through its role in the Tip60 complex [3]. Loss of *dom* function has also been shown to alter the expression of multiple genes [11], one of which was assayed (see below). The results reported here extend these studies, and reveal that *dom* function intersects with the activity of many other chromatin-associated proteins. It is noteworthy that under 22°C conditions, the majority of loci tested did not exhibit an RNAi-mediated LOF phenotype when tested in *Gal4* control crosses, but did synergize with *dom* (26/31 strains = 84%; Tables 1 and 2). Therefore, expression of *dom* RNAi across the dorsal-ventral margin apparently sensitizes the wing to loss of other regulatory inputs. These inputs likely impinge on major cell pathways; as described below, numerous modifier loci have been associated with cell proliferation/cell cycle, cell death and Notch signaling. This is not unexpected given that many of these chromatin component loci are well-described global regulators that localize to a large number of genomic sites [17,15,16].

For example, among the previously-characterized class of chromatin factors [15,16], *CAF1* was the strongest enhancer detected. CAF1 (chromatin assembly factor 1) is a component (NURF55) of the nucleosome remodeling factor complex that regulates cell death and proliferation and Notch signaling [22,23,24]. *CAF1* LOF has been shown to elevate apoptosis in eye discs [23], and we observed elevated levels of cell death across the margin of *C96-Gal4+CAF1* discs, and strong synergism in cell death levels when *CAF1* and *dom* LOF were combined (Fig 3). *PCAF (dGCN5*, P300/CBP-associated factor), another strong enhancer, is a histone acetyltransferase (HAT) that also functions with Notch, and affects cell proliferation in the wing [25]. The *ERR* (estrogen-related receptor) locus enhanced strongly, and it has also been implicated in proliferation [26]; further, we showed previously that another nuclear receptor locus (*EcR*) interacts with *dom* similarly [14]. The *PCNA* (proliferating cell nuclear antigen) locus likely enhances primarily due to effects on efficiency of DNA replication/cell division [27], and we observe no elevation of apoptosis in wing discs deficient for *PCNA* and *dom* (Fig 3). Enhancement via loss of HP1c function may derive from an effect on RNA polymerase elongation [28], thereby generally reducing gene expression levels. HP1c appears to be an atypical, positive transcriptional effector within the HP1 protein group, which typically mark inactive heterochromatic regions of the genome [15]. The *ASF1* (*Anti Silencing Factor*/histone chaperone) and *TOP1* (topoisomerase) enhancers each have effects on transcription and replication [29,30], and ASF1 has also been associated with Notch signaling [31], as well as a *tousled-like kinase* pathway preventing cell death [32]. The latter is consistent with the increased cell death evident in *dom+ASF1* wing discs (Fig 3).

Among the previously-described chromatin factors we also detected three suppressors. *RPD3* (*HDAC1*) encodes a histone deacetylase that has been directly connected to gene repression, and LOF for *RPD3* was associated with elevated expression of Notch pathway targets [31]. Activation of Notch has been demonstrated to suppress apoptosis [33]. These effects could explain the suppressive effect of *RPD3* LOF observed here Table 1, Figs 1 and 3), since an activated form of Notch was shown to rescue the *dom* LOF wing phenotype [14]. *NUP50* encodes a nucleoporin and it is possible that the interaction with *dom* reflects a more general effect on nuclear transport or association of pores with chromatin [16]. The *RYBP* locus (ring and YY1 binding protein) encodes a Zn-finger protein that elicits a wide range of defects during embryonic and imaginal development, including effects on cell division [34].

We also tested a group of 16 loci that were not previously associated with chromatin, but found by DamID to bind specific chromosomal regions [16]. Our assay included 13 of these “chromatin component” (CC) loci and three additional loci that were studied in different contexts, but not as chromatin components (*JIGR1*, *MAF-S*, *TRIP1*) prior to this analysis [16]. As several of these loci are relatively uncharacterized beyond their predicted sequence or physical binding partners, genetic descriptions are limited.

We found that 4 moderate *dom* enhancers, that showed no effect when assayed for LOF alone in control crosses (*CC8*, *JIGR1*, *CC25*, and *CC20*), all contain a MADF (Myb/SANT-like Domain in Adf-1) DNA-binding domain. An additional locus (*CC34*) also encodes this domain, but did not show an interaction. The MADF domain is similar to the SANT domain (a domain found in Dom, as well as ISWI and BRM), and was first identified in the transcriptional activator, Adh transcription factor 1 (Adf-1). Proteins with the MADF domain have been found to act as both activators and repressors [35]. It may be relevant that 4 of the 5 proteins in our study that contain a MADF domain behaved similarly in the assay for *dom* modification (Table 1). For example, LOF for *CC20* affects wing hinge structure [35], as is the case for *dom* LOF, which produces a held out wing phenotype in adults carrying very weak *dom* alleles [2]. Likewise, *JIGR1* was scored among the positives in a screen for genes that were upregulated by RAS expression in hyper-proliferating hemocytes [36]. Although *dom* was not scored in this screen, the role of *dom* in hemocytes is well described [1], thus the function of these loci could intersect in hemocytes. Additionally, the *dom* enhancer *CC9* was also scored as a positive in this screen [36], however *CC9*, which is relatively uncharacterized, does not encode a MADF-containing protein.

A few additional modifiers exhibit other features that indicate an overlap in function with *dom*. The *CC15* locus encodes a Zn-finger protein, and it was detected as a significant enhancer of *dom*. Prior RNAi knockdown studies of *CC15* demonstrated effects on cell cycle progression, increased cell death, and a held out wing phenotype [37]. The CC32 locus was scored as a strong *dom* suppressor. It also encodes a Zn-finger protein that was found in a screen for novel genes involved in hematopoiesis [38]. *MAF-S*, scored as a moderate suppressor of *dom*, has been shown to modulate Myc-induced growth [39]. The remaining four modifiers (*CC4*, *CC7*, *CC24*, *CC28*) encode proteins with known domains that associate with chromatin, and they have been mapped by DamID to regions of active loci (*CC7*, *CC24*, *CC28*) or inactive loci (CC4) [16]; however there is not sufficient information available on these loci to infer a further relationship to *dom*. We do note however a report [11] that the expression of the CC24 transcript is diminished in *dom* mutant larvae, which is consistent with the phenotypic enhancement effect we observe (Table 1). Three of the tested loci did not show significant modification of the *dom* phenotype (*CC34*, *CC30* and *DSP1*), and four additional loci (*HEL25E*, *CC31*, *SIR2*, *TRIP1*) produced highly-penetrant LOF phenotypes in a wild type *dom* background (Table 1, *C96*-*Gal4* column) that precluded scoring a *dom* LOF interaction in adult wings. However, we did examine *CC31* further for an effect on cell death. *CC31* codes for a zinc finger protein, Motif 1 binding protein (M1BP) that is required for cell viability and proliferation [40]. In both the *C96-domR* and *C96-Gal4* crosses *CC31* LOF resulted in 100% wing nicking, and a substantially smaller wing (Table 1, Fig 1). We observed that *C96-Gal4+CC31* wing discs exhibited high levels of apoptosis across the margin, and that even higher levels were apparent when *CC31* was combined with *dom* LOF (Fig 3), indicating again a synergistic interaction between these loci.

Since Dom is a member of the SWI2/SNF2 protein family we also investigated genetic interactions with a set of TRiP RNAi strains that target 12 other family members. The SWI2/SNF2 chromatin remodelers act in numerous contexts, including transcription, replication, DNA repair, recombination and chromosome segregation [41]. We reasoned that Dom could function in related pathways and tissues, or act redundantly with other SWI2/SNF2 proteins; either of these possibilities may be detected through genetic interaction studies. We found that 83% (10/12) of the tested loci elicited a significant modification to the *dom* wing phenotype, whereas only two of these 10 exhibited an RNAi-mediated phenotype in control crosses (Table 2).

The early phenotypic descriptions of *dom* alleles implicated the locus in immunity/hematopoiesis and homeotic gene regulation [1,2]. Four of the modifiers: *ISWI*, *CHD1*, *HEL89B* and *ETL1* affect immunity or hemocyte number [42,43,44], whereas five have been described as homeotic regulators: *ISWI*, *INO80*, *BRM*, *Mi*-*2* and *KISMET* [45,46,2,47,48]. We reported previously that *dom* has a role in Notch signaling [12,14], and *ISWI*, *CHD1*, *BRM* and *KISMET* have also been shown to interact with *Notch* or lead to nicked wings when mutated [49,50,51]. Finally, the role of Dom within the Tip60 complex at double stranded DNA breaks revealed a role in DNA repair through acetylation and exchange of phosphorylated histone H2Ax(v) [3]. *INO80* and *MARCAL1* have also been implicated in repair [41,52,53].

In conclusion, it is perhaps not surprising that a substantial fraction of loci encoding chromatin-associated proteins was found to intersect with *dom* function, as this may reflect the broad realms of function and collaboration among these regulatory genes. As described earlier, Dom has been associated with many fundamental cell processes, including cell proliferation, cell viability/death, autophagy, Notch signaling and both positive and negative gene regulation through its participation in the Tip60 complex. Moreover, LOF for *dom* has been associated with widespread changes in gene activity [11]. The complexity of genetic interactions between *dom* and other loci that intersect with these pathways is illustrated by *Notch*. In some contexts Notch signaling can induce cell proliferation [54] and block cell death [33]. As Dom appears to be a positive effector of Notch signaling [12,4,13,14], these observations are consistent with the original phenotypic descriptions of *dom* alleles [1,2]. However, Dom has also been characterized as a repressor of E2F-responsive loci, and therefore a negative factor in cell proliferation control [10]. Loss of function for *dom* also synergizes with genotypes depressed in autophagy pathway activity [14], and the well-described antagonistic interactions between the autophagy and cell death pathways [55] may add further complexity to these phenotypes. Consequently, interpreting genetic interactions among loci that network with Dom must be done cautiously, since the interactions likely reflect substantial pleiotropic effects.
